# A dataset of multi-level street-block divisions of 985 cities worldwide

**DOI:** 10.1038/s41597-025-04704-7

**Published:** 2025-03-19

**Authors:** Jintong Tang, Liyan Xu, Hongbin Yu, Hezhishi Jiang, Dejie He, Tianshu Li, Wanchen Xiao, Xinying Zheng, Keyi Liu, Yiqin Li, Shijie Li, Qian Huang, Jun Zhang, Yinsheng Zhou, Lun Wu, Yu Liu

**Affiliations:** 1 Institute of Remote Sensing and Geographical Information Systems, School of Earth and Space Sciences, Peking University, Beijing, China; 2 College of Architecture and Landscape Architecture, Peking University, Beijing, China; 3 The Bartlett Centre for Advanced Spatial Analysis, University College London, London, United Kingdom; 4 Academy for Advanced Interdisciplinary Studies, Peking University, Beijing, China; 5 College of Urban and Environmental Sciences, Peking University, Beijing, China; 6 Institute of Geographic Sciences and Natural Resources Research, Chinese Academy of Science, Beijing, China; 7 Stuart Weitzman School of Design, University of Pennsylvania, Philadelphia, PA USA; 8 Global Technical Service Dept, Huawei Technologies, Beijing, China; 9 Global Technical Service Dept, Huawei Technologies, Shanghai, China

**Keywords:** Geography, Interdisciplinary studies

## Abstract

Street-blocks, as basic geographical units for dividing urban space, are widely used in urban planning and statistics. However, the availability and quality of street-block data vary significantly across different countries or regions worldwide. While developed countries tend to have mature urban street-block division systems and corresponding public data, such data in most developing countries are often incomplete or non-existent. Even in countries with available data, the lack of consistent standards for street-block division causes difficulty in international comparative research. To address this gap, we are releasing a new open dataset: *Multi-level Street-block Divisions of 985 Cities Worldwide* (MSDCW), offering a logical, standardized, and user-friendly street-block division system for cities with the estimated population over 500,000 by Demographia from 142 countries or regions, with results at five spatial levels. Validation shows that compared with official datasets, MSDCW offers a reasonable division of urban street-blocks, and is therefore suitable as foundational data for related research. Additionally, researchers can use our method to generate their own street-block division datasets.

## Background & Summary

Street-blocks are basic units for spatial analysis at a crucial geographical scale in various fields such as urban geography^1^, urban morphology^2^, and population geography^3^. They also serve as convenient spatial statistical units for integrating various demographic and socioeconomic data, providing a high spatial resolution foundation for numerous research projects and practical applications^4^. For example, the New York Zoning Resolution includes planning requirements for street-blocks^5^; the U.S. Census uses Census Blocks defined by the Topologically Integrated Geographic Encoding and Referencing (TIGER) system as the basic geographical units for statistics^6^; and the Regulatory Detailed Planning system in China specifies the spatial extent and land use types for street-blocks and parcels^7^. Compared with regular grids that are often used in theoretical analysis and calculation, e.g., square mesh and raster data, irregular grids like street-blocks are usually designed to follow some realistic standards for ease of practicality, while regular grids are not convenient to use in these real-world situations because regular grids do not consider various forms of spatial boundaries, especially roads.

**Fig. 1:**
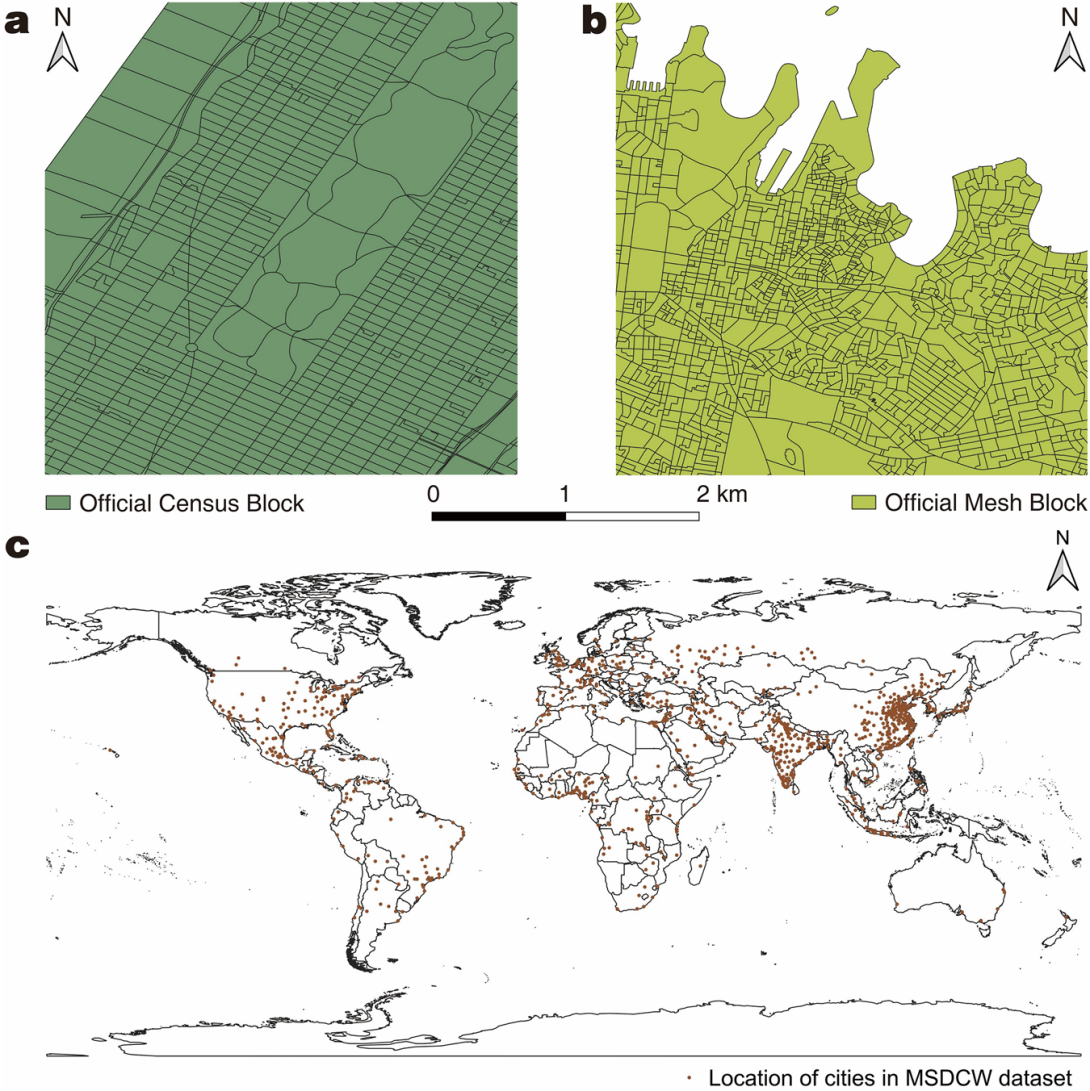
Examples of the official street-block data. (**a**) The Census Blocks in a part of New York, USA. (**b**) Mesh Blocks in a part of Sydney, Australia. Both are on the same scale. (**c**) The distribution of the cities in MSDCW dataset^19^.

However, street-block data’s concept, availability, and quality significantly differ worldwide. In conceptual terms, although street-blocks are usually intuitively understood as space enclosed by geographical features like streets, rivers, and railways, a consistent yet practical definition is to our knowledge absent, due to many methodological obstacles. First, the methods of street-block division vary by country. For instance, the U.S. Census Bureau’s TIGER data is divided into four levels below states: Counties, Census Tracts, Block Groups, and Census Blocks (Fig. 1a) (https://www.census.gov/programs-surveys/geography/guidance/hierarchy.html). In contrast, the Australian Statistical Geography Standard (ASGS) divides areas below states into five levels: Statistical Areas Level 4, Level 3, Level 2, Level 1 (simplified as SA4, SA3, SA2, and SA1, respectively), and Mesh Blocks (Fig. 1b) (https://www.abs.gov.au/statistics/standards/australian-statistical-geography-standard-asgs-edition-3/jul2021-jun2026/main-structure-and-greater-capital-city-statistical-areas). A comparison of Census Blocks in New York, USA, and Mesh Blocks in Sydney, Australia, at the same spatial scope shows differences in scales and shapes of street-block divisions. The differences are partly due to variations in urban spatial structures, but also because of the different methods used to define street-blocks. Second, data availability varies by country. Planning or statistical departments in some countries in the Americas (e.g., USA (https://www.census.gov/geographies/mapping-files/time-series/geo/tiger-line-file.html), Canada (https://open.canada.ca/data/en/dataset/ef70dc3b-1069-4037-9bce-61f47e628a1d), Brazil^8^, and Chile (https://www.ine.gob.cl/herramientas/portal-de-mapas/geodatos-abiertos)), Europe (e.g., UK (https://geoportal.statistics.gov.uk/search?q=OutputAreas&type=featurelayer) and EU countries (https://ec.europa.eu/eurostat/web/gisco/geodata/statistical-units/territorial-units-statistics)), Oceania (e.g. Australia (https://www.abs.gov.au/statistics/standards/australian-statistical-geography-standard-asgs-edition-3/jul2021-jun2026/access-and-downloads/digital-boundary-files) and New Zealand (https://datafinder.stats.govt.nz/data/)), and East Asia (e.g., Japan (https://www.e-stat.go.jp/gis/statmap-search?type=2)) usually publish their street-block division data. However, many other countries lack such data (e.g. some low- and middle-income countries^9^), do not publish it publicly (e.g., mainland China^10^), only allow online browsing (e.g., Egypt (https://proceedings.esri.com/library/userconf/unic17/papers/un_01.pdf)), or restrict access to specific groups (e.g., South Korea requires registration with national ID (https://sgis.kostat.go.kr/view/pss/openDataIntrcn), and some African countries’ census data sets are only available for download to specific institutions (https://guides.library.upenn.edu/EastViewCensusGIS)). Third, the quality of street-block data varies greatly among countries. Developed countries generally have long-term updated and clearly defined street-block data systems (https://www.census.gov/programs-surveys/geography/guidance/hierarchy.html), while developing countries often have low-frequency updates, resulting in poor data quality, especially in smaller cities^10^. These differences make the practicability of similar spatial analysis in different countries vary greatly, with countries lacking these data face challenges in conducting various studies at the street-block level.

To address the issues above, some researchers have designed their own processes for dividing street-blocks for supporting their research. For example, Kırlanhıçoğlu designed the geographical boundaries of census statistical units for Çankaya District, Ankara, Turkey, based on the EU’s NUTS standards^11^; Liu and Long generated and publicly released parcel data for 654 cities in China^10^; Gong *et al*. generated the essenstial urban land use categories data of China^12^, and Chen *et al*. also did so for USA using open big data^13^; Grippa *et al*. produced street-block data for creating urban land use maps in Ouagadougou, the capital of Burkina Faso, and Dakar, the capital of Senegal^14^. However, these ad hoc street-block division schemes may lead to inconsistencies in comparative studies across different countries, resulting in incomparability and even bias^15^. Therefore, both academic research and practices in planning and policy require a universally applicable urban street-block division scheme that are based on reasonable and consistent division methods. Here, we hypothesize that this is feasible because street-blocks, as the basic unit of urban spatial organization, intrinsically exhibit a high degree of regularity and cross-regional consistency in terms of scale and form^16–18^.

Therefore, we have developed a comprehensive street-block division process and thereby released the *Multi-level Street-block Divisions of 985 Cities Worldwide* (MSDCW)^19^. This dataset encompasses 142 countries and regions and 985 urban areas (Fig. 1c) as covered in *Demographia World Urban Areas 17*^*th*^ *Annual Edition*^20^, with each city having five spatial levels of street-block division based on the hierarchy of roads to meet various requirements. The 985 urban areas are selected because their estimated population is larger than 500,000 in 2021 by Demographia^20^, and the population of them is 2.27 billion in total, constituting 51.4% of the population living in cities around the world estimated in 2021^20^. The advantages of this dataset are: (1) The quality of this dataset is basically comparable to that of official data in countries where official division data are available, thus providing a set of high-quality street-block data for countries lacking official datasets, which is able to satisfy the needs of research and management in different cities around the world. (2) Thanks to the uniform division rationale, the dataset ensures overall consistency of the quality of the data across world cities as much as possible, which is helpful for international comparative studies, especially among developed and developing countries.

## Methods

**Fig. 2:**
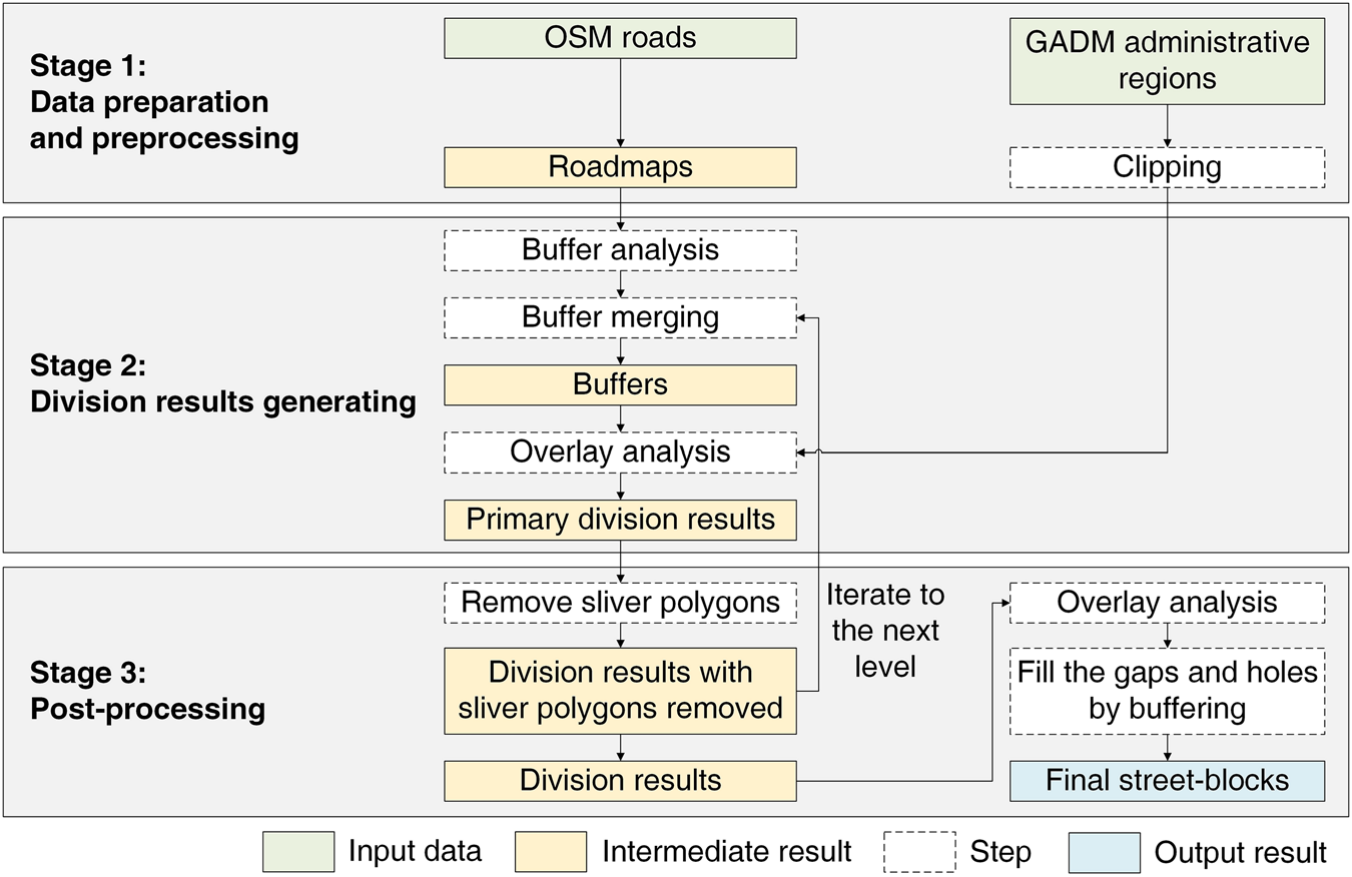
Methodological framework.

**Fig. 3:**
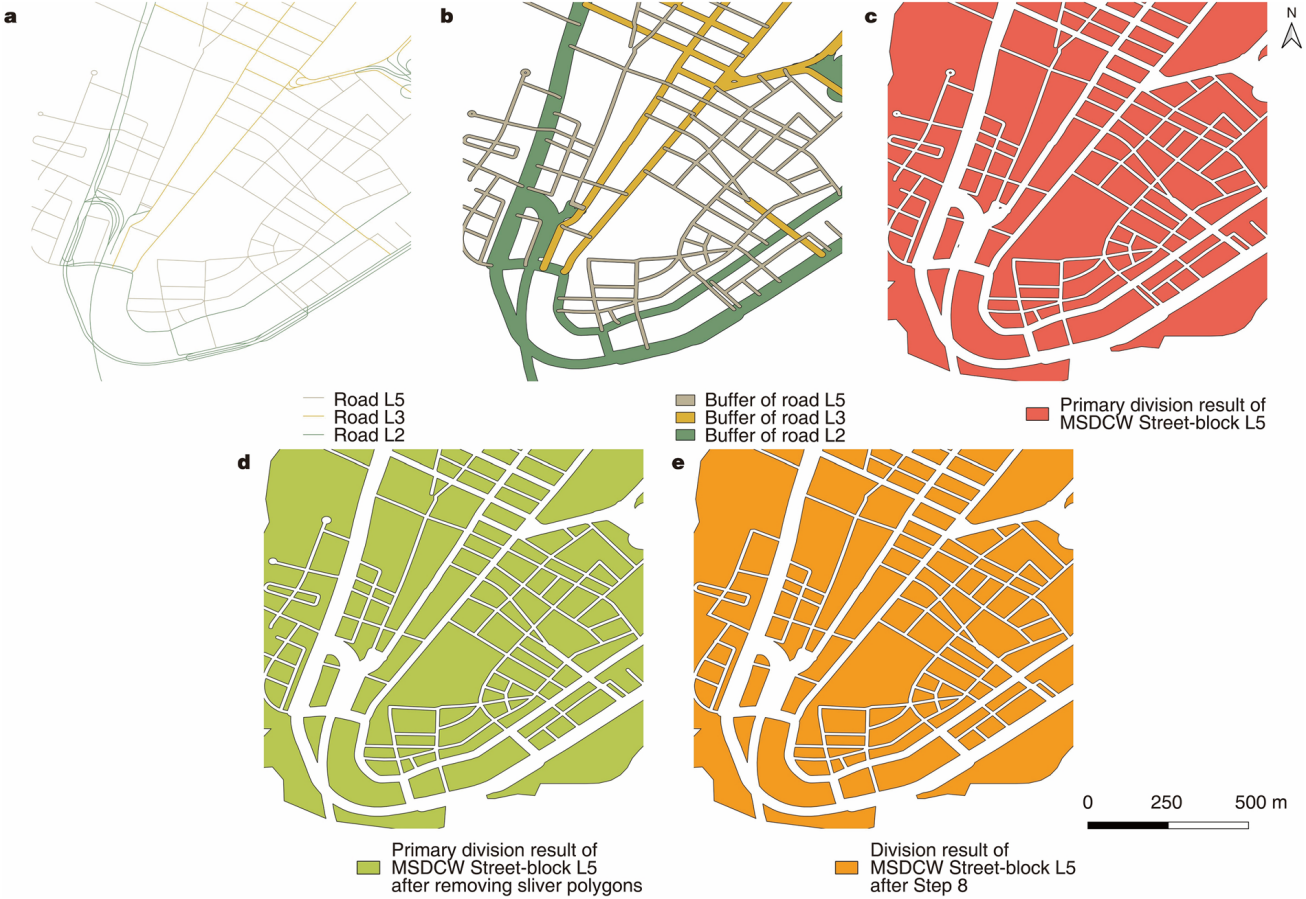
Dataset generation steps. (**a**) Roads at each level; (**b**) Buffers corresponding to roads at each level; (**c**) Primary division result of L5; (**d**) Primary division result of L5 after removing sliver polygons; (**e**) Division result of L5 after Step 8.

The methodological framework for generating the dataset is illustrated in Fig. 2. The entire process contains three stages: data preparation and preprocessing, division results generating, and post-processing. For a clear demonstration of the process, we chose Lower Manhattan in New York, USA, as an example area and illustrated the main steps (Fig. 3).

### Stage 1: Data preparation and preprocessing

**Step 1:** The necessary data were obtained from different sources.

The roads were obtained from OpenStreetMap (OSM) (downloaded from GeoFabrik (https://download.geofabrik.de) for the respective country or region on May 27-28, 2022). OSM is the open dataset with the longest road lengths in the world^21,22^. Its data integrity is high in developed countries^23^ and is gradually improving in developing countries^24,25^. The roads obtained from OSM are lines. We did not perform topology processing (e.g., removing double lines) because it would have introduced more topology issues that would have affected subsequent steps.

The administrative region data were obtained from the Database of Global Administrative Areas (GADM) v3.6 (https://gadm.org/download_world36.html) for street-block division. GADM is a widely used dataset of administrative areas around the world to generate different datasets^26–28^. Given that countries have their claims to their territory and there is no uniform definition of city administrative regions worldwide, and that such regions may also be subject to adjustments, GADM data were only used as a basis for delineating the spatial scope (urban area) of street-block divisions.

The spatial range of division is an adjustable parameter. One possible choice is using “urbanized areas”. However, although urbanized areas can roughly be understood as settlements with higher population densities, the methods of measurement in the literature and the official definitions by countries vary widely^1,29^, encompassing criteria like population statistics, administrative divisions, built environment, economic activities, and even “urban characteristics”^30^. When it is challenging to obtain standard statistical geographic units and the respective population data, remote sensing imagery is widely used for delineating urban areas, such as night lights^31^ or impervious surfaces^32^. Another choice is the jurisdictional ranges of cities, but they can sometimes also be problematic as city-level jurisdictions in different countries may vary greatly in size in both absolute and relative terms. For example, in China, cities include “Province-level City” (*Zhixia Shi* in Chinese), “Prefecture-level City” (*DiJi Shi* in Chinese), and “County-level City” (*Xianji Shi* in Chinese), and they all often cover large rural areas, and are hence with jurisdictions typically much larger than the urban built-up areas^33^. In contrast, city jurisdictions in the United States tend to be smaller, and too often they cover only part of the complete build-up area of a functional city and generally not include rural areas. Besides, U.S. cities may cover multiple counties. For instance, The City of New York consists of five boroughs, each of which is a county of New York State (https://portal.311.nyc.gov/article/?kanumber=KA-02877).

When processing our dataset, we first found jurisdictional regions with the same name in *Demographia World Urban Areas 17*^*th*^ *Annual Edition*^20^. If there are multiple levels of jurisdictional regions in GADM v3.6 with the same name, we selected the lower-level one. If the names of a city in GADM v3.6 and *Demographia World Urban Areas 17*^*th*^ *Annual Edition*^20^ could not be matched directly, we manually selected the correct jurisdictional region in GADM v3.6 as their boundaries. Then we aligned the built-up area of the city (using remote sensing images on Google Map tile layer) with its official jurisdictional range in GADM v3.6 (note that the number of levels of jurisdictional regions are different for different countries, for example, three in China and two in Japan). The basic rule is to keep the complete jurisdictional region for this city, which may include one or more lowest-level jurisdictional regions of the city, and may also include some rural area. But two types of necessary modification were performed.

1. If the lowest-level jurisdictional regions of some countries are too coarse in GADM v3.6, each of them will cover too oversized rural areas. Therefore, we clipped the boundaries to wipe out their rural area.
2. If the built-up area of a city covers its neighboring cities’ jurisdictional range, and the neighboring cities are not in our city list, we added the overlapping lowest-level jurisdictional regions of the neighboring cities to the city we were processing.

Another issue is that *Demographia World Urban Areas 17*^*th*^ *Annual Edition*^20^ shows some “adjacent urban areas”, which means that the built-up area of more than one cities are linked together, e.g., Tokyo-Yokohama, Japan and Guangzhou-Foshan, China. We kept their settings and regarded the jurisdictional regions of each group of them as a whole, and decided whether to edit their boundaries according to the rules above.

### Stage 2: Division results generating

**Step 2:** The highest (default is 1) and lowest levels were set for street-block division. In this dataset, the lowest level was set to 5. Thus, the levels of data obtained in this dataset were sequentially referred to as Level 1, Level 2,…, and Level 5 (simplified as L1, L2,…, L5 hereafter). Note that the division granularity of *higher*-level street-blocks is *coarser*, while that of *lower*-level street-blocks is *finer* (for example, L5 has a finer granularity than L1). The reason for this setting is that our dataset was produced from coarse scale to fine scale by using roads to cut the polygons level by level (see the following steps for detail). Based on the road types given in the OSM road network data (field “fclass”), buffer width for each level of roads and corresponding street-block levels were assigned to produce a table mapping road levels, street-block levels, and road widths.

Like in the case of spatial range designation, the roads of the same functional grade may nominally different in different countries. For example, the *Highway Law of the People’s Republic of China* classifies roads based on their status in the road network into National Highways (*GuoDao* in Chinese), Provincial Highways (*ShengDao* in Chinese), County Highways (*XianDao* in Chinese), and Township Highways (*XiangDao* in Chinese)^34^. The USA classifies roads into Principal Arterial, Minor Arterial, Collector, and Local categories^35^. Meanwhile, OSM’s road classifications include other types such as motorway, trunk, primary, secondary, etc., and the mapping rules between these nominal categories and the official standards of various countries are unknown.

In our dataset, the road levels and their corresponding buffer widths are shown in Table 1. The particular buffer widths were delineated per the rationale of Le Corbusier’s *Modular*, which represents the standard measure for the human body and is thus utilized to define the basic dimensions of the built environment^36^. We therefore set the width of a typical urban road (four motor vehicle lanes, two non-motor vehicle lanes, and two sidewalks) to be 24 meters, and a typical urban residential or rural road (two motor vehicle lanes, without non-motor vehicle lanes and sidewalks) 8 meters, and so forth. These settings are supported by relevant standing standards in various countries. For example, in *Code for Design of Urban Road Engineering* of China, the width of a motor vehicle lane is 3.25 m to 3.75 m (about 11 feet); non-motor vehicle lanes are generally 1 to 2 m wide; and the minimum width of sidewalks along roads is 2 m^37^. In the USA, *A Policy on the Geometric Design of Highways and Streets* prescribes motor vehicle lane widths as 10 to 12 feet (about 3.0 to 3.6 m)^38^. In Britain, *Road Layout Design CD 127*: *cross-sections and headrooms* typically set the lane width at 3.65 to 3.70 m (about 12 feet)^39^. Overall, lane design standards only vary slightly among countries given contextual differences in city terrain and land scarcity, which supports our uniform parameter delineation.

**Table 1:** Mapping parameters used in the released dataset.

Level	Road Type	Road Buffer Width (m)	Sliver Polygon Area Threshold (m^2^)	Buffer width of street-blocks in Street-block Expansion and Street-block Erosion (m)
1	motorway trunk trunk_link motorway_link	24	10,000	42
2	primary primary_link	20	10,000	35
3	secondary secondary_link	16	1,000	28
4	tertiary tertiary_link	12	100	22
5	residential	8	100	16

However, no matter how these parameters are set, the road data used for dividing *lower*-level street-blocks should include those for dividing *higher*-level street-blocks (for example, the roads used for dividing L5 include those for dividing L1 to L5).

Here, the generation of L5 is used as an example in the following text unless specified otherwise.

**Step 3:** Starting with the highest level as set in Step 2, the corresponding level of roads (lines in OSM data) were extracted (Fig. 3a).

**Step 4:** Buffers of corresponding widths for roads at each level (Fig. 3b) were generated according to the Table 1 (set in Step 2). Then, the buffers of road networks at all levels were merged. Some topological errors of the roads could be fixed through this operation. For example, when a buffer was performed to the roads, some roads that should be linked but were not could be connected by such an operation.

**Step 5:** An overlay analysis was performed using the buffers generated in Step 4 to obtain *the primary division result*. Figure 3c shows the primary division result in the case area.

### Stage 3: Post-processing

For the primary division results of each level:

**Step 6:** A sliver polygon analysis was performed. Loops and ramp residuals in non-edge areas of the primary division results where the area under certain thresholds (Table 1) were removed. On the one hand, the distribution of areas of street blocks in cities of the world shows a power law (with power exponent -2)^16^. On the other hand, since the street-block areas of different cities are different due to their planning tradition, applying a uniform standard to all cities is not optimal, as it may preserve too small street-blocks in some cities while removing those that should be preserved in others. Therefore, we decided to keep smaller thresholds for different levels to keep as much street-blocks as possible by using the powers of 10 as thresholds. We also set different thresholds for different levels, because different levels of street-blocks were produced using different categories of roads. Higher-level roads are relatively sparser, while lower-level roads are relatively denser, resulting in higher thresholds for higher-level street-blocks than those with lower levels. Figure 3d shows a comparison of the primary division results after removing sliver polygons.

**Step 7:** The primary division results of L1 after removing sliver polygons were input into Steps 4-5 to calculate the division results of L2. Then, the primary division results of L2 after removing sliver polygons were input into Steps 4-5 to calculate the division results of L3. This process was repeated from higher to lower levels, sequentially outputting the division results for street-blocks at all levels.

**Step 8:** More processing operations can be performed based on actual needs. First, the division results at each level were further refined using data on administrative division boundaries to obtain the division results. Second, the internal gaps and holes of the street-blocks caused by the multiple parallel lines (double lines in most cases), dead-end roads, and the ramps of overpasses were filled (Fig. 3e) to get more regular shape for the street-blocks. This operation has three sub-steps. (1) Hole Filling: The wkt string of a polygon with holes is composed of multiple polygons. The information of the polygons corresponding to the holes are always located at the back of the wkt string. Therefore, the holes were filled by only preserving the information of the first polygon in each wkt string. (2) Street-block Expansion: *Positive* buffers (widths shown in Table 1) were performed to the street-blocks first to fill the gaps first, so the dead-end roads were filled by this operation. (3) Street-block Erosion: Buffers with *the opposite number* of the widths in the Street-block Expansion sub-step (that is, this buffer width is *negative*) were performed on these filled street-blocks to restore their size and remove their untidy sides. In Street-block Expansion and Street-block Erosion sub-steps, considering the possible double lines or even multiple lines for the road data, we set these buffer widths smaller than the multiple of the number of the parallel roads, because if we set this width too large, the distances of the parallel roads may be not as wide as the buffer widths, and the shape of the street-blocks we produced after the erosion sub-step may be changed inconsistently from that before this sub-step.

## Data Records

The dataset^19^, its corresponding codes, and the list of 985 cities are available at: https://figshare.com/articles/dataset/MSDCW_Dataset_and_Code/26021314. In the released dataset, the data for each country is stored in its respective ZIP file. Each file contains shapefiles at each level for cities in the country or region. Since the OSM data we used were obtained on May 27-28, 2022, this dataset shows the situation at that time. The filename for each shapefile data is “(Country and Region)_(City), (Abbreviation of province or state, only for some countries) _L(MSDCW^19^ street-block level, in Arabic numerals).(filename extension, such as shp, dbf, shx, etc.)”, for example, “China_Wuhan, HUB_L1.shp” represents the MSDCW^19^ L1 data for Wuhan City in Hubei Province, China. Each shapefile contains only one field “index”. This field provides a unique ID for the street-blocks at that level in the city, starting from 0 and increasing in steps of 1. The geographical coordinate system for all data is WGS 84, and no planar projection has been applied. We have provided a list with the names of the 985 cities together with the street-block data^19^, which can be also downloaded in the link we provided. To show modification type (1) and (2) of city boundaries in Step 1 more clearly, we marked it as “1” and “2” in the “modification” column of the city list.

## Technical Validation

### Quantitative comparison with official division data

For cities with official division data available, we selected several cities based on the regions published by the World Bank^40^. However, the data from the countries of South Asia is not available to our knowledge.

We list the data sources and their corresponding time in Supplementary Table S1. We tried to align MSDCW dataset^19^ with the official datasets at the same time point and the same spatial range as much as possible. In terms of time, the official dataset may not be updated frequently, and some countries do not release the historical version of their official datasets, so there are still some time differences between the MSDCW dataset^19^ and the official dataset for some cities. As for the spatial range, we preserved complete street-blocks in the comparable range as much as possible. Specifically, we pick out the street-blocks in the official dataset of each city that intersect with those in MSDCW dataset^19^ L1, the coarsest level, of each city to retain as much street-blocks as possible. Due to different geometric accuracies of the datasets, some discrepancies at the boundaries might occur. To maintain comparability and simplify the analysis in different cities, these discrepancies had been kept, inevitably introducing some biases to the results. The geographical coordinate systems of all official datasets had been converted to WGS 84.

We selected multiple indicators for a quantitative comparison. The principles for selecting indicators are as follows. First, they must encompass the intrinsic properties of geometric shapes as much as possible; Second, they must be able to directly compare the consistency between datasets. Meanwhile, as Louf and Barthélemy pointed out that the area and shape factors of urban street-blocks reflect their overall characteristics and thus allow for typological classification^16^, we also chose these two types of indicators for comparison. Eventually, we opted to use the following four indicators:

Indicator 1 and 2: the average area ($\bar{A}$) and the coefficient of variation (${CV}$) of street-blocks at a certain level, which measure the overall level of area and scale consistency (i.e., dispersion) of street-blocks at each level of both datasets. A higher $\bar{A}$ indicates a larger average area of street-blocks, and vice versa. A higher ${CV}$ indicates greater dispersion in the area of street-blocks (i.e., the areas of individual street-blocks are more uneven), and vice versa. We believe that the size of $\bar{A}$ does not imply quality difference, but a lower area coefficient of variation indicates better division results. Here, the area of street-blocks is calculated in km².

Indicator 3: the average Shape Index^41^ (abbreviated as ${SI}$) for each street-block, denoted as $\overline{{SI}}$, which assesses the complexity of the shapes of street-blocks at each level. Using a square as a reference, the equation for calculating the ${SI}$ of each street-block is:

1 $${SI}=\frac{E}{4\sqrt{A}}$$

where $E$ represents the perimeter of the street-block, and $A$ is the area of the street-block. The ${SI}$ of a square is 1. The larger $\overline{{SI}}$ is, generally the more complex the shapes of the street-blocks are; conversely, the simpler. Therefore, a smaller $\overline{{SI}}$ indicates better division results.

Indicator 4: the boundary consistency (${BC}$) value, which directly compare the consistency between the MSDCW^19^ dataset and the official dataset. Let ${\mathcal{P}}$ denote the set of intersecting polygons of two street-block dataset, and |${\mathcal{P}}$| denotes the number of the elements in ${\mathcal{P}}$, then definition of the ${BC}$ value is:

2 $${BC}=\frac{{\sum }_{p{\mathscr{\in }}{\mathcal{P}}}\max \left({{AP}}_{A},{{AP}}_{B}\right)}{\left|{\mathcal{P}}\right|}$$

In Eq. (2), ${{AP}}_{A}$ shows the percentage of the intersecting polygon $p\in {\mathcal{P}}$ to the corresponding street-block of dataset A, while ${{AP}}_{B}$ shows that of dataset B. The maximum of ${{AP}}_{A}$ and ${{AP}}_{B}$ shows how consistent the two corresponding street-blocks from the two datasets are, because the two datasets have inclusion relationship with each other which should be simultaneously considered. We believe that there are two cases that can reflect better agreement. First, two polygons are similar in area and have a high degree of intersection. Second, there is a containment relationship of a large polygon to a small polygon, a higher result can also be obtained after taking the maximum value. Then the ${BC}$ value is calculated by averaging the maximum values for all intersecting polygons in ${\mathcal{P}}$. The minimum and the maximum values of ${BC}$ are 0 and 1, respectively. The bigger it is, the more consistent the two datasets are, and the better the division of MSDCW^19^ of this city is when the official division plan is viewed as a baseline.

When calculating the above indicators, for data from developed countries, the ${BC}$ value is calculated pairwise between each level of official data and each level of MSDCW^19^ data. For Johannesburg-Pretoria and Dar es Salaam, since we can only access official data at one level, we only use this level of official data to calculate the indicators. In calculating the geometric properties related to each indicator, the geographical coordinate system of the official dataset is first converted to WGS 84. Then, both the official and MSDCW^19^ data’s projection coordinate systems are converted to their respective UTM 6-degree zone projection coordinate systems. This conversion allows for the scale to be converted into metric lengths, facilitating the display of urban forms at the same scale.

**Fig. 4:**
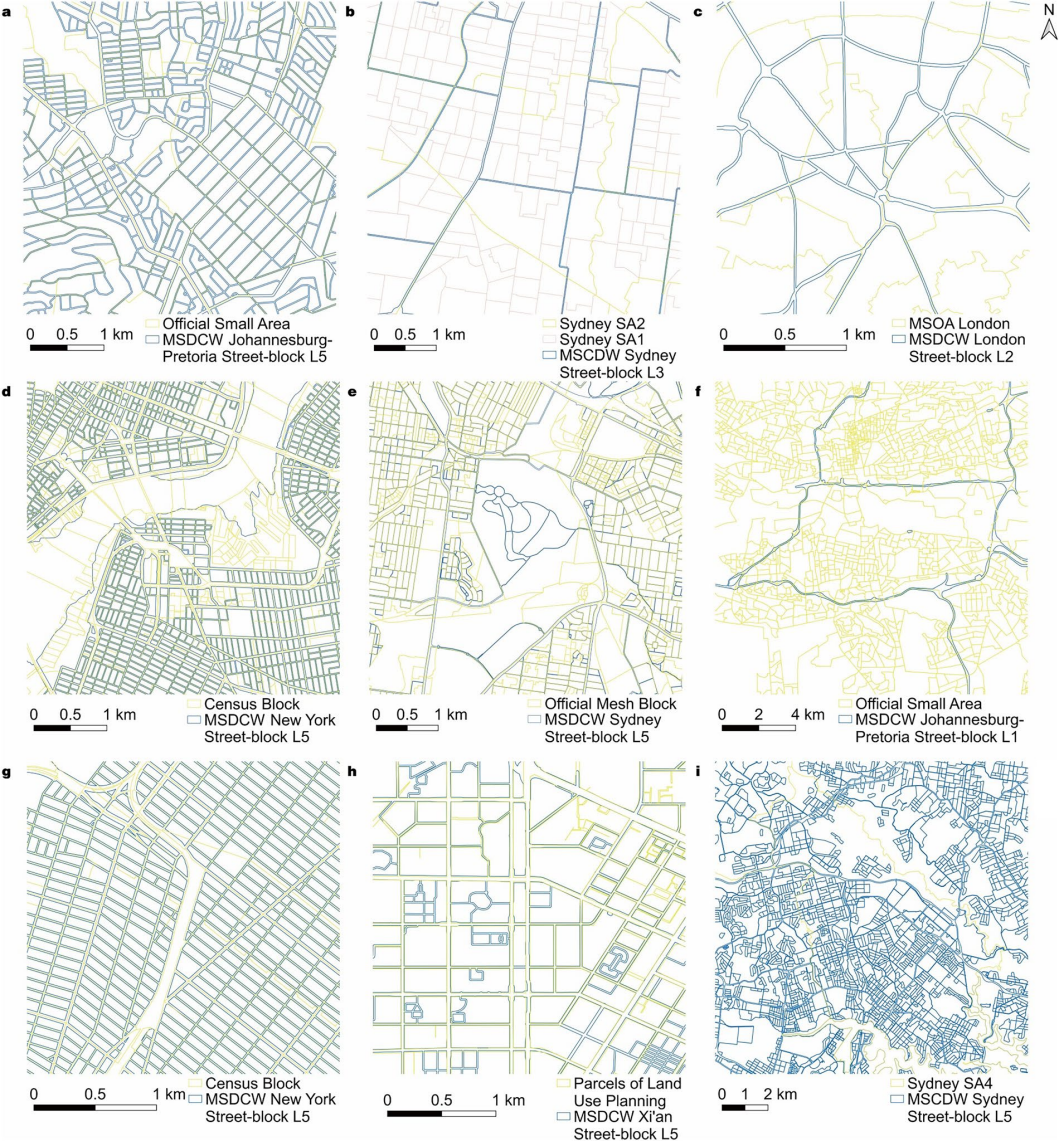
Comparison of official street-block data with the MSDCW dataset^19^. (**a**) Comparison of official census data with L5 for Johannesburg-Pretoria; (**b**) Comparison of SA1 and SA2 with L3 for Sydney; (**c**) Comparison of MSOA with L2 for London; (**d**) Comparison of Census Blocks with L5 for New York; (**e**) Comparison of Mesh Blocks with L5 for Sydney; (**f**) Comparison of official Small Areas with L1 for Johannesburg-Pretoria; (**g**) Another comparison of Census Blocks with L5 for New York; (**h**) Comparison of parcels of land use planning with L5 for Xi’an; (**i**) Comparison of SA4 with L5 for Sydney.

The results for the three indicators, $\bar{A}$, ${CV}$, and $\overline{{SI}}$ are shown in Tables 2–4, respectively. Figure 4 shows some comparisons of the official datasets and different levels of MSDCW^19^ datasets. From the perspective of self-comparison for the MSDCW dataset^19^ in each city, first, it generally finds levels that are roughly equivalent to official data in all three indicators. This is due to the basic consistency of the division logic between our dataset and the official datasets from various countries, which is based on road divisions. Although we did not find related literature for South Africa, the visualization demonstrates such consistency (Fig. 4a). Second, we have provided reasonable supplements for scales not covered by the official dataset, such as the Sydney L3 data scale, which is between the official SA1 and SA2 (Fig. 4b).

**Table 2:** The indicators of $\bar{A}$(km^2^) for street-blocks from the MSDCW dataset^19^ and official datasets of different levels in selected cities.

City	Levels of MSDCW^19^					Levels of Official Data														
	L1	L2	L3	L4	L5															
Nanjing	81.395	20.750	6.333	1.978	1.047	Parcels of land use planning														
						0.383														
Xi’an	136.747	39.526	10.658	3.424	1.285	Parcels of land use planning														
						0.166														
Tokyo	54.779	12.837	5.861	1.015	0.044	City/Rural Blocks				Basic Unit Blocks						Enumeration Districts				
						0.325				0.042						0.010				
Sydney	52.460	14.544	4.801	1.600	0.130	SA4	SA3					SA2			SA1					Mesh Block
						869.487	253.613					31.103			0.694					0.088
Singapore	12.830	2.788	1.438	0.836	0.152	Planning Areas		Subzones					Land Use Layers					Cadastral Land Parcels		
						14.266		2.363					0.007					0.005		
London	39.369	6.289	3.475	1.187	0.116	MSOA					LSOA						OA			
						5.313					0.929						0.165			
Mexico City	49.069	11.433	3.994	1.180	0.039	AGEM					AGEB						Manzanas			
						93.321					0.609						0.010			
Buenos Aires	241.200	60.089	5.645	1.654	0.034	Radio Censales														
						0.428														
Cairo	34.084	13.436	5.170	1.410	0.053	Shyakha and Qurya														
						28.489														
New York	21.882	8.012	2.244	0.906	0.065	Counties			Census Tracts					Block Groups					Census Blocks	
						1150.588			2.957					0.983					0.078	
Johannesburg-Pretoria	149.379	22.318	5.951	2.241	0.070	Small Areas														
						0.492														
Dar es Salaam	116.717	74.064	30.664	8.497	0.067	Hamlets/Enumeration Areas														
						0.130

**Table 3:** The indicators of ${CV}$ for street-blocks from the MSDCW dataset^19^ and official datasets of different levels in selected cities.

City	Levels of MSDCW^19^					Levels of Official Data														
	L1	L2	L3	L4	L5															
Nanjing	0.672	0.552	0.375	0.240	0.179	Parcels of land use planning														
						0.420														
Xi’an	0.494	0.262	0.144	0.086	0.053	Parcels of land use planning														
						0.205														
Tokyo	0.245	0.149	0.152	0.084	0.022	City/Rural Blocks				Basic Unit Blocks						Enumeration Districts				
						0.251				0.119						0.060				
Sydney	0.431	0.336	0.239	0.156	0.048	SA4	SA3					SA2			SA1					Mesh Block
						0.723	0.480					0.167			0.028					0.048
Singapore	0.537	0.451	0.339	0.277	0.135	Planning Areas		Subzones					Land Use Layers					Cadastral Land Parcels		
						0.955		0.367					0.040					0.064		
London	0.596	0.421	0.443	0.400	0.181	MSOA					LSOA						OA			
						0.403					0.306						0.218			
Mexico City	0.351	0.232	0.159	0.094	0.021	AGEM					AGEB						Manzanas			
						1.057					0.131						0.208			
Buenos Aires	0.642	0.440	0.213	0.143	0.034	Radio Censales														
						0.143														
Cairo	0.396	0.246	0.162	0.094	0.018	Shyakha and Qurya														
						0.117														
New York	0.271	0.360	0.287	0.269	0.104	Counties			Census Tracts					Block Groups					Census Blocks	
						0.598			0.061					0.035					0.057	
Johannesburg-Pretoria	0.730	0.487	0.424	0.349	0.069	Small Areas														
						0.110														
Dar es Salaam	0.521	0.461	0.350	0.274	0.032	Hamlets/Enumeration Areas														
						0.130

**Table 4:** The indicators of $\overline{{SI}}$ for street-blocks from the MSDCW dataset^19^ and official datasets of different levels in selected cities.

City	Levels of MSDCW^19^					Levels of Official Data														
	L1	L2	L3	L4	L5															
Nanjing	1.237	1.233	1.218	1.183	1.209	Parcels of land use planning														
						1.368														
Xi’an	1.658	1.375	1.311	1.259	1.240	Parcels of land use planning														
						1.319														
Tokyo	1.583	1.325	1.269	1.200	1.183	City/Rural Blocks				Basic Unit Blocks						Enumeration Districts				
						1.215				1.716						1.177				
Sydney	1.741	1.462	1.311	1.243	1.283	SA4	SA3					SA2			SA1					Mesh Block
						2.425	1.926					1.440			1.192					1.182
Singapore	1.458	1.250	1.220	1.327	1.621	Planning Areas		Subzones					Land Use Layers					Cadastral Land Parcels		
						1.497		1.234					1.382					1.530		
London	1.316	1.217	1.208	1.204	1.248	MSOA					LSOA						OA			
						1.638					1.587						1.454			
Mexico City	1.499	1.388	1.407	1.374	1.240	AGEM					AGEB						Manzanas			
						1.379					1.255						1.263			
Buenos Aires	1.846	1.480	1.320	1.503	1.083	Radio Censales														
						1.141														
Cairo	1.404	1.392	1.447	1.619	1.305	Shyakha and Qurya														
						1.312														
New York	1.509	1.406	1.315	1.277	1.192	Counties			Census Tracts					Block Groups					Census Blocks	
						1.265			1.191					1.198					1.212	
Johannesburg-Pretoria	1.391	1.330	1.258	1.231	1.307	Small Areas														
						1.198														
Dar es Salaam	1.375	1.365	1.318	1.300	1.161	Hamlets/Enumeration Areas														
						1.144

From a cross-city comparison perspective, the scale, area dispersion, and shape regularity of MSDCW^19^ data of cities from developed countries (i.e., Tokyo, Sydney, Singapore, London, and New York) are generally finer than those in developing countries (i.e., Nanjing, Xi’an, Mexico City, Buenos Aires, Cairo, Johannesburg-Pretoria, and Dar es Salaam). The higher OSM data quality leads to finer division of street-blocks in developed countries, while considering the relative lack of basic city data in developing countries^23^, it is acceptable for data of the same level in these countries to be coarser. This implies that street-block divisions from various countries are comparable when following similar division logics, and that our dataset can provide effective supplements of different granularities for regions where official data is scarce or unavailable.

There are still certain levels of discrepancies between the MSDCW dataset^19^ and the official ones, some reflecting the former’s shortcomings, while others are caused by complex reasons. On the one hand, since sliver polygons might not have been deleted completely, the degree of dispersion in the area of street-blocks (represented by ${CV}$) for data of similar scales in some cities is relatively high. For example, the normally sized street-blocks with very small street-blocks coexist in London L2 and MSOA data (Fig. 4c). This issue could be addressed by further filtering out sliver polygons. On the other hand, the complexity of shapes in some cities is also somewhat high. This is due to multiple factors: (a) coastal street-blocks in the MSDCW^19^ retain the shape of the coastline, while official datasets simplify them with straight lines (like New York) (Fig. 4d); (b) official division data tends to ignore smaller roads, thus producing more square results (Fig. 4e); (c) in suburbs, higher-level roads themselves tend to be curved, making the shapes of MSDCW^19^ division results more irregular (Fig. 4f). These being said, overall, it can be considered that the division results of MSDCW^19^ street-blocks are at least on par with official data and superior in some cases.

The ${BC}$ values for the cities are shown in Table 5, with 140 comparison pairs in total. Overall, the ${BC}$ values of 108 comparison pairs are higher than 0.7, with that of 87 comparison pairs greater than 0.8 and 56 comparison pairs greater than 0.9, covering each city in our validation, regardless of developed and developing countries, or the regions they belong to. This indicates that the dataset obtained from our unified division logic has good consistency with the official datasets. The following explanations account for these high matching values. First, the division logic of street-blocks is consistent (i.e., all based on roads), such as New York’s Census Blocks^42^ with L5 (Fig. 4g), and Xi’an’s Parcels of land use planning with L5 (Fig. 4h). Second, larger street-blocks can completely encompass lower-level street-blocks (note that this encompassing relationship can occur between each other), for example, Sydney’s SA4 with L2~L5 (Fig. 4i shows a comparison of Sydney’s SA4 with L5). This caused the high average ${BC}$ values for L1 and L5, because L1 data are large enough to cover polygons from finer levels of official dataset, while L5 data are small enough to be covered by those from coarser levels of official dataset.

**Table 5:** The indicator of ${BC}$ for street-blocks from the MSDCW dataset^19^ and official datasets of different levels in selected cities.

City	Levels of Official Data	Levels of MSDCW^19^					Average for Levels of Official Data	Average for City
		L1	L2	L3	L4	L5		
Nanjing	Parcels of land use planning	0.967	0.950	0.889	0.821	0.815	0.888	0.888
Xi’an	Parcels of land use planning	0.989	0.969	0.921	0.855	0.815	0.910	0.910
Tokyo	City/Rural Blocks	0.878	0.744	0.646	0.547	0.880	0.739	0.831
	Basic Unit Blocks	0.979	0.953	0.932	0.878	0.625	0.873	
	Enumeration Districts	0.980	0.956	0.937	0.888	0.635	0.879	
Sydney	SA4	0.582	0.783	0.881	0.948	0.994	0.838	0.788
	SA3	0.427	0.597	0.757	0.887	0.985	0.731	
	SA2	0.590	0.489	0.516	0.697	0.947	0.648	
	SA1	0.954	0.918	0.860	0.758	0.713	0.841	
	Mesh Blocks	0.974	0.956	0.932	0.896	0.666	0.885	
Singapore	Planning Areas	0.566	0.723	0.830	0.888	0.969	0.795	0.872
	Subzones	0.810	0.700	0.731	0.794	0.925	0.792	
	Land Use Layers	0.992	0.981	0.972	0.963	0.887	0.959	
	Cadastral Land Parcels	0.984	0.962	0.950	0.938	0.873	0.941	
London	MSOA	0.647	0.413	0.397	0.471	0.794	0.544	0.632
	LSOA	0.845	0.627	0.551	0.442	0.597	0.612	
	OA	0.944	0.840	0.789	0.669	0.449	0.738	
New York	Counties	0.827	0.873	0.956	0.975	0.996	0.925	0.850
	Census Tracts	0.799	0.671	0.601	0.639	0.955	0.733	
	Block Groups	0.895	0.818	0.721	0.680	0.936	0.810	
	Census Blocks	0.975	0.953	0.922	0.899	0.901	0.930	
Mexico City	AGEM	0.547	0.769	0.889	0.953	0.994	0.830	0.852
	AGEB	0.922	0.771	0.700	0.593	0.833	0.764	
	Manzanas	0.996	0.989	0.984	0.976	0.862	0.961	
Buenos Aires	Radio Censales	0.967	0.941	0.842	0.711	0.786	0.849	0.849
Cairo	Shyakha and Qurya	0.728	0.634	0.637	0.785	0.971	0.751	0.751
Johannesburg-Pretoria	Small Areas	0.975	0.941	0.882	0.797	0.843	0.888	0.888
Dar es Salaam	Hamlets/Enumeration Areas	0.986	0.980	0.972	0.943	0.749	0.926	0.926
Average for Level		0.847	0.818	0.807	0.796	0.836	0.821	N/A

As for discrepancies, especially those where the ${BC}$ values are lower than 0.6 at specific cities and division levels. The main factor for this phenomenon is that there may be specific considerations in the official street-block division of the area. For example, the average ${BC}$ value of London is lower than that of other cities, and Fig. 4c shows the inconsistency. This is because that the criteria for designing of MSOA includes factors like population size and social homogeneity^43^, which is similar to the standard of OA^44^.

### Additional qualitative comparison of division data among countries in the world

**Fig. 5:**
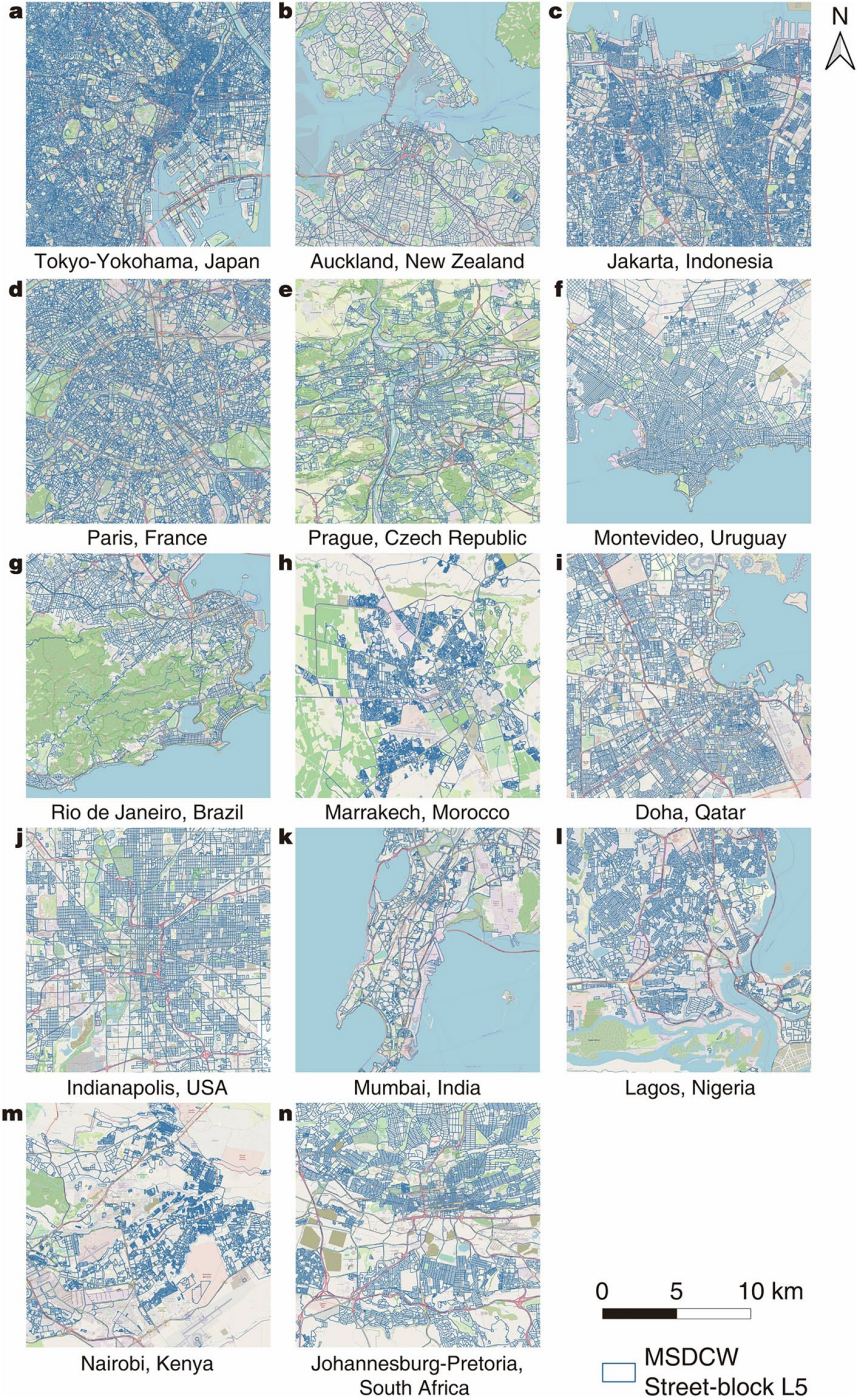
Comparison of OSM (base map) and MSDCW dataset^19^.

Many cities around the world either do not have official street-block division schemes or we are unable to access them, and there are also differences in the completeness of OSM data across different countries. In our methods, the division of street-blocks is directly determined by the local road conditions. Roads are important components of urban morphology, and the differences in urban morphology often relate to factors including local natural conditions, cultural traditions, and government management^45^. Therefore, we have selected typical cities, also based on the regions published by the World Bank^40^, to showcase their OSM base maps and MSDCW^19^ division results. This aims to help users grasp an overview of the data in different parts of the world. Considering the representativeness in terms of geographical areas, urban morphology, level of economic development, and completeness of OSM data, case cities are selected as shown in Table 6. Figure 5 displays the comparison between the OSM base maps and MSDCW^19^ division results for these cases, with the MSDCW^19^ division results illustrated at L5.

**Table 6:** Cities selected to show the data quality.

Region by World Bank	City Name
East Asia and Pacific	Tokyo-Yokohama, Japan
	Auckland, New Zealand
	Jakarta, Indonesia
Europe and Central Asia	Paris, France
	Prague, Czech Republic
Latin America and Caribbean	Montevideo, Uruguay
	Rio de Janeiro, Brazil
Middle East and North Africa	Marrakech, Morocco
	Doha, Qatar
North America	Indianapolis, USA
South Asia	Mumbai, India
Sub-Saharan Africa	Lagos, Nigeria
	Nairobi, Kenya
	Johannesburg-Pretoria, South Africa

The street-block division results of these cities reflect not only the quality differences of OSM data in various regions but also the different urban textures formed by the influences of terrain, history, culture, and planning systems. For example, the Greater Tokyo Area, centered in Tokyo, Japan, is the most populous metropolis in the world, characterized by high population density, dense blocks, and small block scales. Auckland, the largest city in New Zealand, is located in the hilly area on the east coast of the North Island, resulting in an uneven terrain, thus the city road networks are irregular, leading to irregularly shaped street-blocks. Today’s street-block pattern of Paris, France, results from transformations led by Haussmann in the mid-19th century, with the city roads being straight and blocks relatively small^46^. Prague, the capital of the Czech Republic, is an ancient city with a smaller block scale that has preserved the traditional urban street pattern well, resulting in more irregular street-block shapes. The street-block patterns in Montevideo, Uruguay, and Indianapolis, USA, are very regular. The former was built by Spanish colonizers who adopted the then-prevailing small-block rules in Spanish Colonial America, hence the predominance of square street-blocks^47^. The latter, located in the plains of the northeastern United States, due to the implementation of the *Land Ordinance of 1785*, had its land divided into standard rectangles, making today’s city street-blocks quite uniform^48^. Doha, the capital of Qatar, developed rapidly in the 1960s with oil drilling, demonstrates a clear distinction between the old and new towns^49^. The old town streets are with an irregular pattern typical of old Islamic cities^50^, while those in the new town areas are significantly more modernist and orderly. The similar pattern is also observed in Marrakech, Morocco. Finally, the historical old town of the city center of Mumbai, India, and the informal settlements of Nairobi, Kenya, Rio de Janeiro, Brazil, and Lagos, Nigeria, show similar issues in their division results: although the actual road density and population density are high, due to the lack of detailed internal road data for dividing street-blocks, coarser street-block division results were yielded in the MSDCW dataset^19^.

The above two sections have verified and discussed the MSDCW^19^ division results, comparing with official division data in three cities and OSM base maps in cities around the globe. Overall, for cities worldwide, the basic logic of the division algorithm used by the MSDCW^19^ dataset is generally reasonable, and this consistent division logic ensures that the dataset largely matches the actual urban form. Therefore, the dataset has good usability and comparability, supplements street-block scales not covered by official data, and can serve as a universal division scheme to support research of various purposes.

## Usage Notes

The dataset may not be the optimal street-block division scheme for users, or they should use the data with caution, under the following circumstance:

1. Since the *Demographia World Urban Areas 17th Annual Edition*^20^ only includes cities with populations over 500,000, this dataset lacks coverage for smaller cities.
2. OSM is a crowdsourced dataset that anyone can edit, and its data is typically in a state of continuous updates. The dataset was created using the nearest OSM data available to that time (2022), instead of the latest OSM data (2024) by the time the dataset was released due to the necessary production timeline. Some new roads have been built after 2022, especially in the rapid urbanizing countries, but this dataset only represents the situation in 2022. However, we believe that this does not significantly impact the overall data quality, for the following reasons. First, systematic surveys have shown that OSM’s road data coverage worldwide had already exceeded 80% until 2017, with relatively complete road network data available in both developed countries and some developing countries^23^. Therefore, the street-block dataset based on data as of 2022 can be considered to have a basic level of quality assurance. Second, in the real world, once roads are built, they rarely undergo changes (such as demolition or rerouting)^51,52^. Generally, changes in road datasets only occur when new roads are constructed, or emergence of existing roads which were not vectorized before due to technical limitations (for example, roads in forests or deserts may have low visibility in remote sensing images). Changes in both scenarios tend to be marginal as compared to the stock road data^53^. Third, in the Technical Validation section, we included a comprehensive coverage of cities in different geographic regions for comparison, in order to demonstrate the generalizability and differences of OSM data across regions with different level of qualities. Results show that in cities in different regions of the world, our dataset can reflect the texture characteristics of the city, including the differences in the characteristics of urban construction in different periods.
  However, some issues of OSM data are still unavoidable, which we advise users take cautions. First, as the OSM data is constantly being updated, the data version used will never be the absolute latest given the time required for production regardless of the time the most recent OSM data is selected for the creation of the street-block dataset. Second, official datasets from various countries are updated much less frequently than the OSM data. Even the most frequently updated datasets, such as the TIGER data from the United States, are usually to the most updated only on an annual basis. Therefore, discrepancies between MSDCW^19^ and official data are also unavoidable. Third, as shown earlier, the limitations of this dataset primarily stem from the variation in OSM data quality across different regions. On the one hand, there is a significant difference in the quality of OSM data between developed and developing countries^23^, with street-blocks in developing countries typically having larger area sizes shown in our experiments. On the other hand, within the same country, there are also notable differences in OSM data quality between city cores and peripheries^54^. As the results show, periphery urban regions often have larger and more irregularly shaped street-blocks. This is partly due to the morphological nature of cities, and yet partly due to the relative lack of road data compared to city cores.

## Supplementary information

Supplementary Table S1

## Data Availability

The code we used to produce this dataset can be accessed together with the dataset^19^ and the list of cities at https://figshare.com/articles/dataset/MSDCW_Dataset_and_Code/26021314.
